# Anomalous Left Coronary Artery Connected to the Pulmonary Artery in a 15-Year-Old Girl: Case Report and Discussion on Secondary Prevention of Sudden Death

**DOI:** 10.1155/2021/7198667

**Published:** 2021-08-31

**Authors:** Jérémy Laïk, Virginie Fouilloux, Philippe Aldebert, Linda Koutbi, Jérôme Hourdain, Philippe De Swardt, Fabrice Tiger, Anne Bellemain-Appaix, François Bernasconi, Laurent Jacq

**Affiliations:** ^1^Department of Cardiology, Antibes Juan-les-Pins Hospital, France; ^2^Department of Cardiac Surgery, La Timone Children Hospital, France; ^3^Department of Cardiology, La Timone Children Hospital, France; ^4^Department of Reanimation, Antibes Juan-les-Pins Hospital, France

## Abstract

*Background*. Anomalous left coronary artery connected to the pulmonary artery (ALCAPA) is a rare congenital heart disease. Adaptive development of sufficient heterocoronary collaterality in the newborn may allow survival to a later age. In older children or adults, malignant ventricular arrhythmias can reveal the disease. *Case Report*. A 15-year-old girl was referred to the local hospital after a resuscitated out-of-hospital cardiac arrest. CT scan and coronary angiography revealed an ALCAPA. Direct aortic reimplantation of the left coronary artery was performed. Postoperative ECG monitoring showed short episodes of nonsustained ventricular tachycardia. Transthoracic echocardiography and cardiac MRI revealed subendocardial fibrosis of the anterolateral papillary muscle. Beta-blockade therapy was initiated at first intention. After hospital discharge, the patient reported several fainting without loss of consciousness. Considering sudden death nonrelated to effort, episodes of nonsustained ventricular tachycardia, and areas of myocardial fibrosis, the patient underwent subcutaneous cardioverter-defibrillator implantation. 6-month follow-up is satisfactory without clinical or rhythmic abnormalities. *Discussion*. Indication for surgical correction of ALCAPA is well defined, but rhythmic secondary prevention after resuscitated cardiac arrest is less consensual. Cardiac MRI is an essential tool in the identification of a potential rhythmic substrate and should be taken into account in the discussion of a preventive cardioverter-defibrillator implantation.

## 1. Introduction

Anomalous left coronary artery connected to the pulmonary artery (ALCAPA) syndrome is a rare congenital coronary anomaly with an incidence of 1 in 300000 live births [1] and accounts for approximately 0.25–0.5% of all congenital heart diseases [2]. The high pulmonary vascular resistance at birth explains the conserved antegrade perfusion in the abnormally connected left coronary artery and therefore the absence of early neonatal symptoms. As pulmonary vascular resistance declines, the left coronary blood flow becomes retrograde and may result in myocardial infarction with heart failure. Without surgery, prognosis is appalling with a mortality rate of 90% within the first year of life [3]. More rarely, adaptive development of sufficient collaterality from the right coronary artery may allow survival to a later age and sometimes to adulthood. Clinical manifestations may then include subclinical ischemia or sudden death due to ventricular arrhythmias.

## 2. Case Presentation

A 15-year-old girl was referred to the local hospital following an out-of-hospital cardiac arrest. She was sitting by a swimming pool when she passed out. Resuscitation maneuvers were initiated immediately, and two external electric shocks were delivered by the semiautomatic defibrillator on ventricular fibrillation. She had no history of heart disease, but her parents had noticed exertional dyspnea.

**Figure 1:**
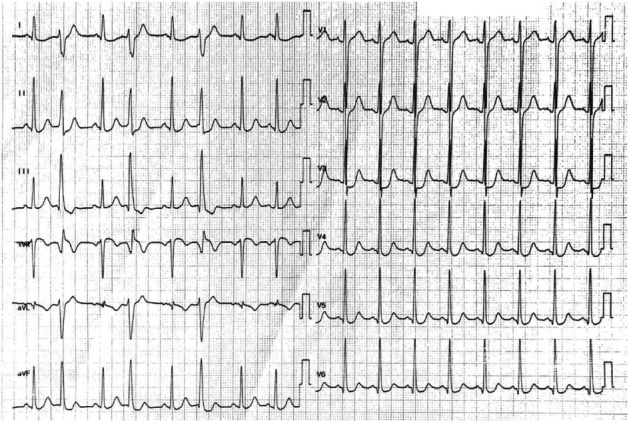
Post-resuscitation electrocardiogram.

**Figure 2:**
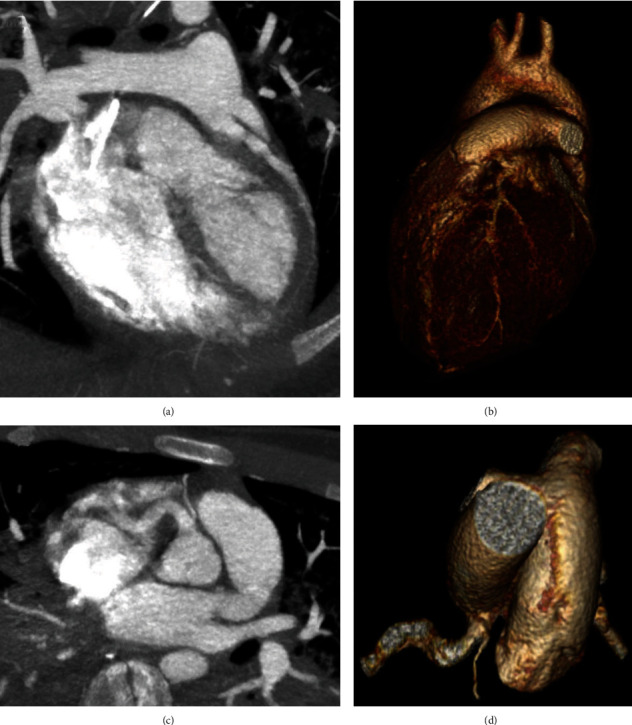
Computed tomography. Three-dimensional multiplanar and volume rendering reconstructions. (a, b) Left coronary artery connected to the pulmonary trunk; (c, d) right coronary artery (and its marginal branch) connected to the aorta.

**Figure 3:**
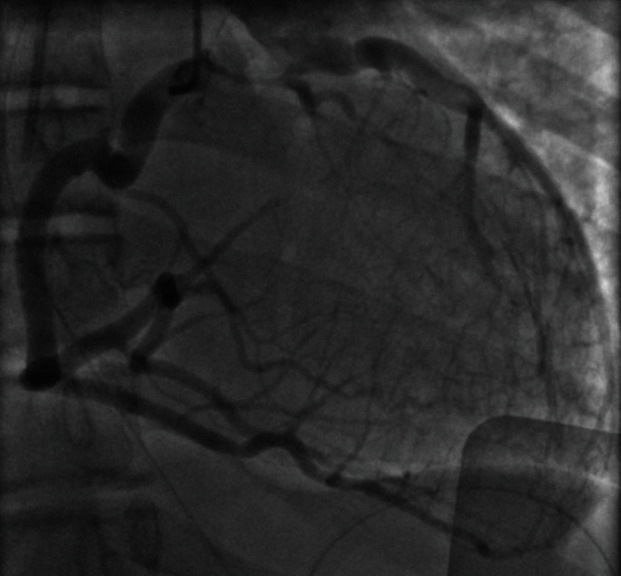
Coronary angiogram. Dilated right coronary artery with left-to-right shunt towards the left coronary artery connected to the pulmonary trunk; note the important collateral network.

In the intensive care unit, hemodynamics was stable without inotropic support. High-sensitivity troponin T was 240 ng/L (reference value < 14 ng/L) with no evidence for acute myocardial ischemia. 12-lead electrocardiogram (ECG) showed apicolateral ST depression with negative T-waves and ventricular bigeminy (Figure 1). Transthoracic echocardiography revealed an undilated left ventricle with moderately impaired systolic function and mild mitral regurgitation. The left coronary artery was not seen at the left aortic sinus. Increased echodensity of the anterolateral papillary muscle and the chordae tendineae was noticed. Chest CT scan showed the presence of an ALCAPA with a compensatory dilated right coronary artery (RCA) (Figure 2). Coronary angiography demonstrated the left-to-right shunt, from the normally implanted RCA towards the left coronary artery and into the pulmonary trunk, through an important network of collaterals (Figure 3). The patient was transferred to the referral center for urgent corrective surgery.

**Figure 4:**
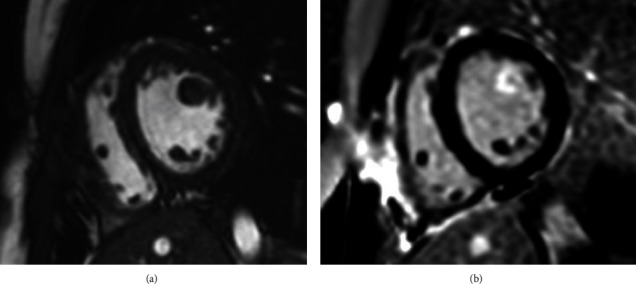
Cardiac MRI—short-axis views. (a) Cine acquisition: note the hypertrophy of the anterolateral papillary muscle; (b) phase-sensitive inversion recovery (PSIR) sequence: late gadolinium enhancement (LGE) of the anterolateral papillary muscle.

Direct aortic reimplantation of the left coronary artery to the aorta was successfully performed. 24 h electrocardiographic monitoring revealed episodes of nonsustained ventricular tachycardia. Postoperative cardiac MRI showed an undilated left ventricle (end-diastolic left ventricular volume 82 mL/m^2^) with preserved systolic function (ejection fraction 63%). Morphological analysis and phase-sensitive inversion recovery sequences found hypertrophy of the anterolateral papillary muscle with late gadolinium enhancement, suggesting areas of subendocardial cicatricial fibrosis (Figure 4). The patient was discharged home twenty days after surgery on bisoprolol 2.5 mg once daily.

One month after hospital discharge, the patient reported several fainting without loss of consciousness. Control coronary angiography revealed a satisfactory operative result with no anastomotic stenosis of the left coronary artery. Given the sudden death at rest, episodes of nonsustained ventricular tachycardia, presence of myocardial fibrosis, and persistent fainting, the patient underwent subcutaneous cardioverter-defibrillator (S-ICD) implantation. Six-month follow-up is satisfactory without clinical or rhythmic abnormalities.

## 3. Discussion

Surgical correction of ALCAPA is the rule and is one of the main emergencies in congenital heart surgery. Different technical approaches have been suggested. Currently, restoration of 2-coronary circulation is considered the gold standard, via direct aortic reimplantation of the left coronary with its pulmonary artery button [3, 4]. Most recent surgical series demonstrated a good postoperative course with low early mortality and a 20-year survival of almost 98% [5]. If the anatomical anomaly has been correctly corrected, long-term ischemia may be responsible for persistent myocardial reshuffle that could be arrhythmogenic. If so, this substrate deserves to be identified and taken into account in the secondary prevention approach, especially if the clinical presentation was a cardiorespiratory arrest.

Shivalkar et al. showed on intraoperative samples that the chronically hypoperfused myocardium is remodeled but theoretically viable, with a postoperative recovery potential [6]. However, histological analysis shows up to 50% cellular fibrosis and there are no published studies after surgery, which raises the question of true reversibility. Current cardiac imaging modalities allow routine noninvasive assessment of myocardial fibrosis. Transthoracic echocardiography is the first-line examination and guides the diagnosis by showing an increased echodensity of the endocardium [7]. Localization is variable and can involve the papillary muscles that may appear hypertrophied [8, 9]. Cardiac MRI is the reference test and looks for late gadolinium enhancement that may also involve the mitral papillary muscle [10, 11]. Browne et al. showed that areas of late enhancement in ALCAPA correspond to areas of microscopic fibrosis [12]. 10-year MRI follow-up after surgery shows that between 65% and 71% of patients keep an abnormal enhancement which, even if generally not very extensive, suggests incomplete myocardial recovery [13, 14].

The long-term clinical significance of these enhancement zones in ALCAPA is not clearly defined, but it is reasonable to believe that they are a potential arrhythmogenic substrate. Indeed, the presence of a myocardial scar is known to serve as a substrate for malignant ventricular arrhythmias. From an electrophysiological point of view, fibrosis promotes the formation of reentry circuits at risk of degenerating into ventricular fibrillation but also creates electrical heterogeneity and automaticity changes that induce fatal arrhythmias [15, 16]. In ALCAPA, ventricular arrhythmias can be secondary to acute local ischemia induced by the coronary steal, but also to a reentry circuit around an infarcted area or to electrical instability due to endocardial fibrosis [17]. In our patient, the occurrence of sudden death at rest argued for a scar-related mechanism and was an additional argument for a preventive implantable cardioverter-defibrillator (ICD).

To date, indication of an ICD for secondary prevention of sudden death in ALCAPA is not consensual. Not all reported cases of cardiac arrest secondary to ALCAPA have been implanted [18]. Nevertheless, implantations have often been motivated by MRI identification of myocardial fibrosis [19–22]. In addition to an identified myocardial scar, the decision-making process should look for other risk factors for sudden death such as severe left ventricular systolic dysfunction, episodes of nonsustained ventricular tachycardia in Holter-ECG or on exertion, or a inducible ventricular arrhythmia at programmed ventricular stimulation if this was performed [23]. Indication for implantation must be balanced against the absence of reported appropriated shocks on implanted ICDs, the necessary risk of complications after ICD implantation in young people regardless of the approach, and the absence of established causality between fibrosis in ALCAPA and long-term rhythmic risk.

Subcutaneous ICD provides an attractive approach in the prevention of sudden death in young patients with congenital heart disease. This technology avoids the frequent lead-related complications of the epicardial or endocavitary material [24, 25]. The infection rate is comparable to endocavitary system but remains low and at lower risk of causing extraction surgery [26]. Inappropriate shocks represent the main complication and are often related to T-wave oversensing [25, 26]. The pre-implantation process must therefore ensure that the patient does not require pacing, and that ventricular sensing is performed correctly in one of the device vectors. Due to the absence of antitachycardia pacing for termination of ventricular tachycardia, management may include antiarrhythmic therapy and possible ablation procedures to treat organized arrhythmias. Recent studies suggest efficacy and complications comparable to the transvenous device in congenital heart disease [26]. Long-term data will confirm whether this approach remains safe and reliable in this very specific population.

## 4. Conclusion

The management of ALCAPA is first and foremost surgical but must also consider the electrophysiological consequences and the potential arrhythmogenic substrate. MRI identification of scar fibrosis is an important element to take into account in the decision-making process for secondary prevention. The S-ICD is an interesting approach in young subjects with congenital heart disease, but preliminary screening has to be rigorous. Scarcity of the pathology makes hardly imaginable comparative studies of prevention strategies, so the successive description of cases may help to enhance the literature on this subject. In the meantime, the discussion of secondary prevention should be taken on a case-by-case basis within a tertiary congenital cardiology center.

## Abbreviations

ALCAPA: Anomalous left coronary artery connected to the pulmonary artery
ECG: Electrocardiogram
RCA: Right coronary artery
MRI: Magnetic resonance imaging
S-ICD: Subcutaneous implantable cardioverter-defibrillator.
